# Electron transfer of extremophiles in bioelectrochemical systems

**DOI:** 10.1007/s00792-022-01279-8

**Published:** 2022-10-12

**Authors:** Miriam Edel, Laura-Alina Philipp, Jonas Lapp, Johannes Reiner, Johannes Gescher

**Affiliations:** 1 Institute of Technical Microbiology, Hamburg University of Technology, Hamburg, Germany; 2 Karlsruhe Institute of Technology, Engler-Bunte-Institute, Karlsruhe, Germany

**Keywords:** Extremophiles, Microbiology, Bioelectrochemical systems, Exoelectrogens, *c*-type cytochromes, Anode interaction, Cathode interaction

## Abstract

The interaction of bacteria and archaea with electrodes is a relatively new research field which spans from fundamental to applied research and influences interdisciplinary research in the fields of microbiology, biochemistry, biotechnology as well as process engineering. Although a substantial understanding of electron transfer processes between microbes and anodes and between microbes and cathodes has been achieved in mesophilic organisms, the mechanisms used by microbes under extremophilic conditions are still in the early stages of discovery. Here, we review our current knowledge on the biochemical solutions that evolved for the interaction of extremophilic organisms with electrodes. To this end, the available knowledge on pure cultures of extremophilic microorganisms has been compiled and the study has been extended with the help of bioinformatic analyses on the potential distribution of different electron transfer mechanisms in extremophilic microorganisms.

## Introduction

Microorganisms can catalytically interact with anode and cathode surfaces in bioelectrochemical systems (BES). This enables a direct or indirect transfer of electrons between the biocatalyst and the bioelectrochemical reactor system (Logan 2009; Lovley and Nevin 2013; Beblawy et al. 2018). The interaction between biology and electrode material can occur either by planktonic organisms using organic or inorganic electron shuttles or by organisms that are organized in the form of biofilms using the electrodes as active growth substratum. Typically, the latter mode of interaction allows for a more efficient electron transfer that is foremost limited by the catalytically available electrode surface and the number of microorganisms that can actively participate in electron transfer (Edel et al. 2019).

Microbe-anode interaction is the best studied mode of catalysis in BES. In these interactions the microorganisms catalyze the direct conversion of chemical into electrical energy by using the anode surface as terminal electron acceptor of their respiratory chain. The anode is used as surrogate for insoluble environmental electron acceptors like ferric or manganese oxides. In addition, also soluble electron acceptors such as oxygen can be replaced by anodes if the organisms are able to transport their electrons to the cell surface. The interaction is possible since the terminal reductases interact rather unspecifically with electron accepting surfaces that are characterized by a redox potential above a certain threshold of around − 200 mV versus standard hydrogen electrode (SHE) (Prokhorova et al. 2017). The adaptation of the enzymes is most likely due to the evolutionary selection pressure that asked for a solution to reduce a very abundant electron acceptor that occurs in the form of minerals with different forms and shapes and not as a molecule being specifically characterized by its dimensions and electrochemical properties (Beblawy et al. 2018).

While the microbe-anode interaction exploits millions of years of environmental selection pressure and seems to be ubiquitously distributed, interaction of microbes with insoluble electron donors does not seem to be as common. Still, multiple lines of evidence suggest geothermal electricity generation in deep hydrothermal systems. Nevertheless, so far we do not have characterized isolates from these field sites but Pillot et al. could at least show enrichment data for cathodic growth of inocula taken at a deep-sea hydrothermal chimney (Nakamura et al. 2010; Pillot et al. 2021). Nevertheless, a direct interaction of isolated extremophiic microorganisms with cathodes has some biotechnological potential as it could offer the possibility to use autotrophic organisms and an electrical current as starting points for a carbon dioxide-based biotechnology. The direct usage of cathodic electrons would provide a cheap and sustainable electron donor that can be produced with a much lower energy input compared to hydrogen. Several studies revealed that the electron export machinery of some organisms that can interact with anodes can function bidirectionally which enables the import of electrons (Dumas et al. 2008; Ross et al. 2011). Still, organisms that are naturally interacting with anodes are often not the best suited catalysts for cathodic applications as they do not thrive autotrophically. On the other hand, for some organisms interacting with cathodes, it is still not clear whether they use electrons or in situ produced hydrogen as electron donor (Philips 2020). A fact that might not necessarily be a pitfall regarding application.

Many reports focused on the biochemistry of model organisms thriving in bioelectrochemical reactors under mesophilic conditions (Philipp et al. 2020). The aim of this review is to summarize our biochemical knowledge on extremophilic organisms interacting directly with anodes and cathodes. Therefore, we will analyze only experimental work conducted with pure cultures as this allows to draw clear conclusions on biochemical pathways operating in these organisms.

## The biochemistry of microbial electron transfer to anodes

### Extended electron transport pathways based on multiheme *c*-type cytochromes

**Fig. 1:**
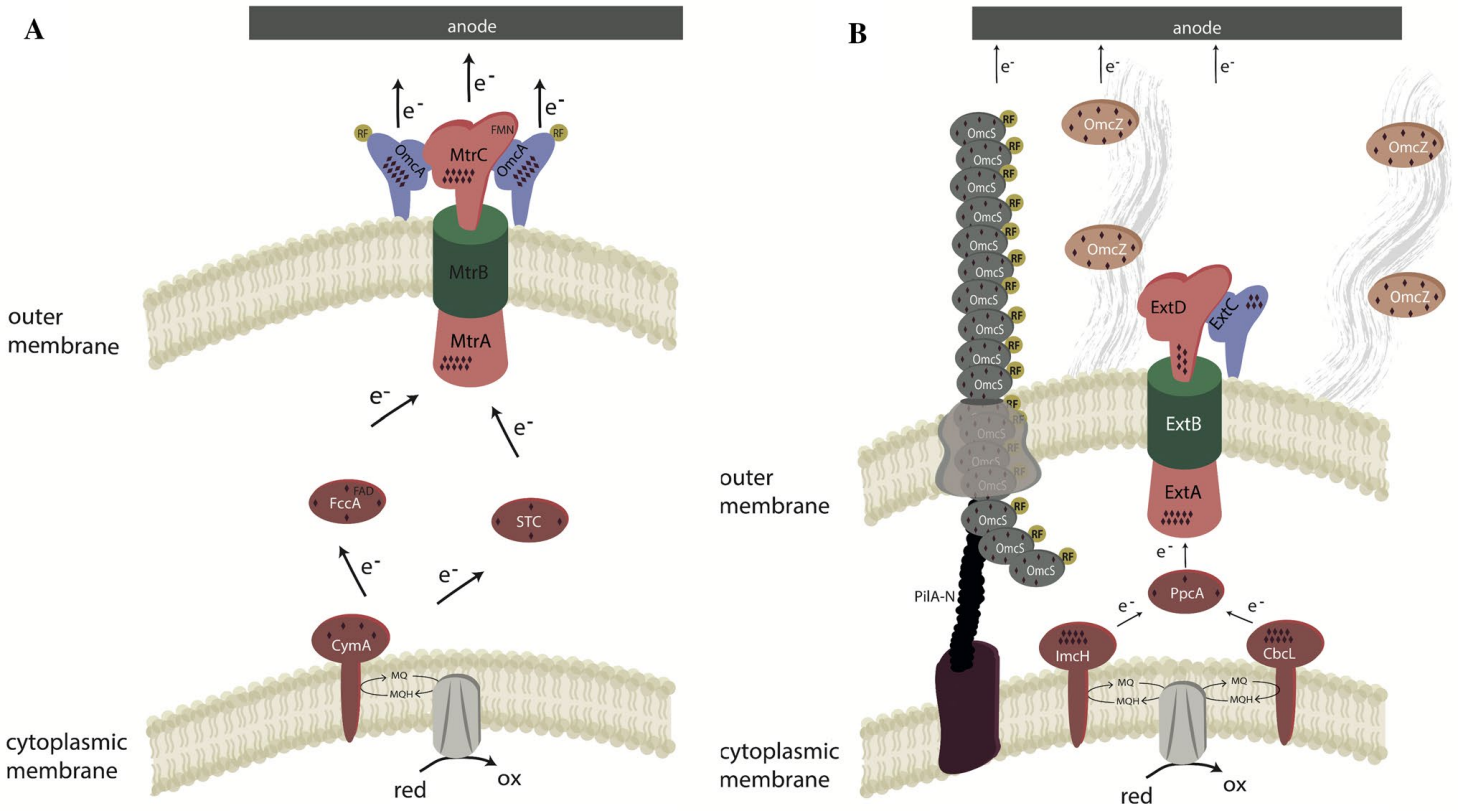
Scheme of the extracellular electron transfer pathways in *S. oneidensis* (**A**) and *G. sulfurreducens* (**B**). Diamonds indicate the number of heme cofactors in the respective *c*-type cytochromes. Scheme modified from Philipp et al. (2020)

Most of our knowledge on the direct transfer of electrons to anode surfaces stems from studies using mesophilic organisms and will be used for a comparison to known strategies under extremophilic conditions. *Shewanella oneidensis* and *Geobacter sulfurreducens* are the mesophilic model organisms for an extended electron transfer chain in Gram-negative organisms. Electron transfer in both organisms is mainly dependent on a network of *c*-type cytochromes spanning the distance from the cytoplasmic membrane to the cell surface (Fig. 1) (Richter et al. 2012; Methé et al. 2003; Romine et al. 2008). *S. oneidensis* uses the tetraheme *c*-type cytochrome CymA to catalyze electron transfer between the quinone pool and the periplasm (McMillan et al. 2012). The equivalents to CymA are the nonaheme *c*-type cytochromes ImcH and CbcL in *G. sulfurreducens*. Interestingly, they are expressed by the organism in a redox potential dependent manner. If the redox potential of the insoluble electron acceptor is at or below -0.1 V vs SHE the organism will operate with CbcL while it will use ImcH above potentials of 0.1 V vs SHE (Zacharoff et al. 2016). Both organisms use soluble periplasmic cytochromes to bridge the gap of the periplasm that is too wide to allow for direct electron hopping (Lloyd et al. 2003; Fonseca et al. 2013). Electron transfer through the outer membrane is catalyzed in both organisms via electron conduits that consist of heterotrimeric complexes of an integral β-barrel protein and *c*-type cytochromes on either side of the membrane. While *S. oneidensis* has again one essential trimeric complex for anode reduction (MtrABC), *G. sulfurreducens* can express several different conduits of which ExtABCD seems to be the most important in BES (Hartshorne et al. 2009; Otero et al. 2018). The electron transport chain in *S. oneidensis* ends with the two decaheme cytochromes MtrC and MtrA that form a loosely attached complex. Interestingly, the terminal reductases in both organisms contain in addition to the heme groups also a flavin as further cofactor that accelerates one electron transfer via the formation of semiquinones (Okamoto et al. 2013, 2014a, 2014b; Xu et al. 2016). Compared to *S. oneidensis*, *G. sulfurreducens* developed further strategies to extend electron transfer beyond the dimensions of a single cell within biofilms. First studies reported on the production of conductive type IV pili by the organism that would extend the dimensions of direct electron transfer to several micrometers in length. The molecular reason for pili conductivity remains controversial, although it was also shown that these type IV pili are periodically decorated by *c*-type cytochromes. Recently, Wang and colleagues and Filman and colleagues revealed that *G. sulfurreducens* produced also pilus like structures that are built by cytochromes as monomers (Wang et al. 2019; Filman et al. 2019). Apparently, the export of these polymeric structures is based on the type IV pilus production machinery (Gu et al. 2021). Hence, it seems possible that the conductive structures build by *G. sulfurreducens* are not type IV pili and that the observed correlation between the presence of genes for type IV pilus production and formation of conductive pili might be the use of the same production machinery for both structures. Even though both of the mentioned organisms are mesophilic heterotrophs, it seems that the same kind of pathway could operate in oligotrophic anammox bacteria that can couple the anaerobic oxidation of ammonium to the reduction of anode surfaces instead of the reduction of NO_2_ (Shaw et al. 2020). Although, this pathway for extracellular electron transfer seems to be the major system operating in Gram-negative anode reducing organisms, it was so far not found in isolated thermophiles.

### Flavoprotein-based extracellular electron transfer in Gram-positive organisms represents a widely distributed strategy also in extremophilic organisms

**Fig. 2:**
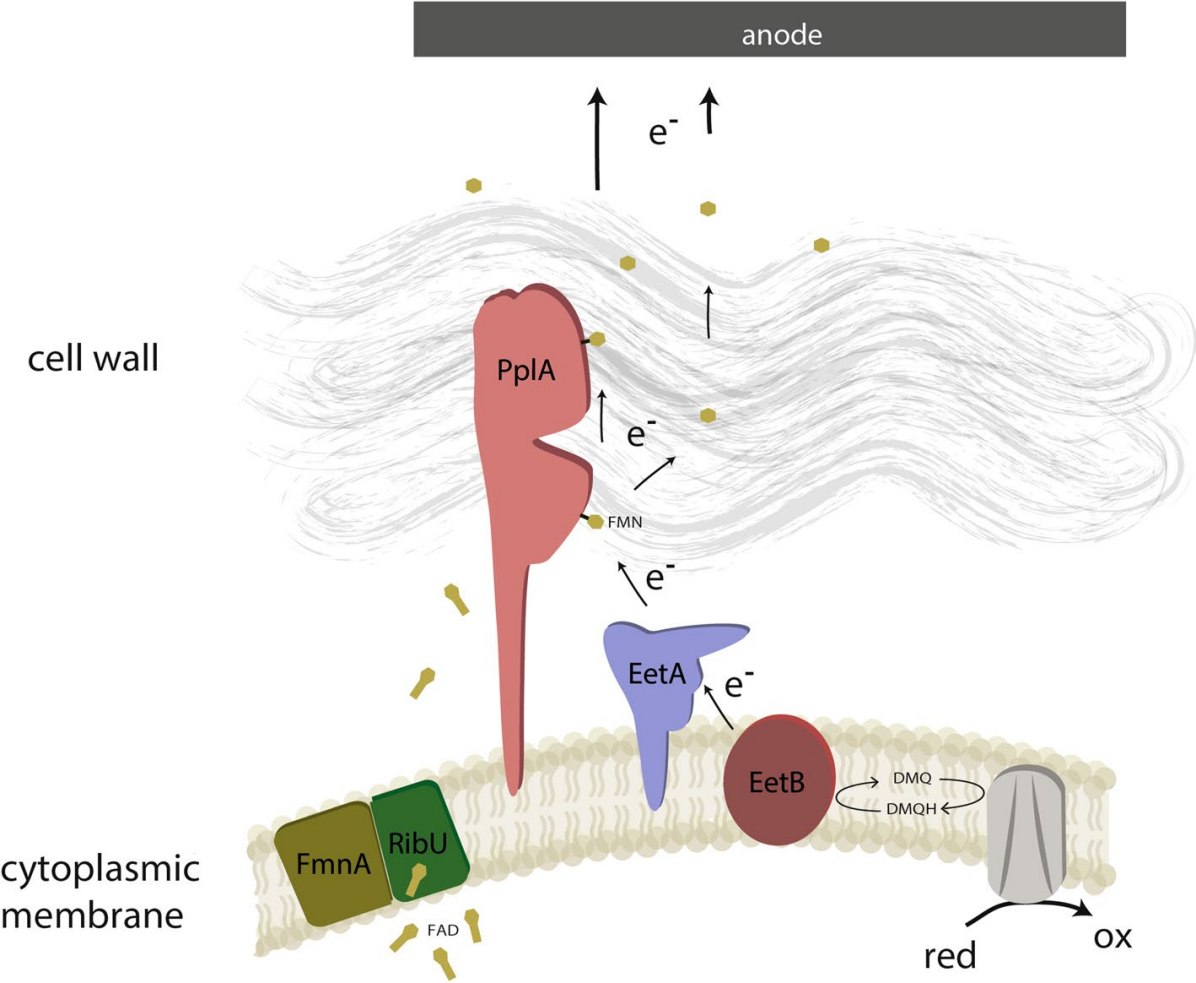
Model of the extracellular electron transfer in *L. monocytogenes*. Scheme modified from Light et al. (2018, 2019)

Years after major discoveries in the field of the biochemistry of extracellular electron transfer to anodes in Gram-negative microorganisms it became clear that another strategy developed in mesophilic Gram-positive organisms that is independent of *c*-type cytochromes. Light and colleagues could show for *Listeria monocytogenes* that electron transfer rates to anode surfaces with comparative rates to *S. oneidensis* can proceed via a polyflavinated lipoprotein as terminal reductase (Light et al. 2018, 2019). This protein is the end of a short electron transport chain involving an NADH dehydrogenase and a dimethylmenaquinone as membrane integral electron shuttle (Fig. 2). The authors searched for the necessary genes for the electron transfer pathway using a transposon screen and revealed a gene cluster containing two genes necessary for methylmenaquinone synthesis (*dmkAB*), a gene for the NADH dehydrogenase (*ndh2*), two genes encoding the flavin transferase (*fmnAB)* necessary for flavinylation of the terminal reductase PplA which is also encoded in the same cluster (*pplA)*. Moreover, two further genes are encoded in the locus which could so far not be assigned to a specific function (*eetAB*). The corresponding genes could be found also in organisms thriving in thermophilic habitats like *Caldanaerobius spp.* and *Thermoanaerobacter spp.* as well as halophilic habitats like *Halolactibacillus* spp. and *Halothermothrix* spp. (Light et al. 2018). A similar mechanism might also be in place in a recently described current producing thermophilic *Geobacillus* strain that can use lignocellulose as carbon and electron source (Shrestha et al. 2020). Hence, it seems that this pathway of cytochrome-independent extracellular electron transfer is by far more adaptable regarding different habitats compared to the above-described cytochrome pathways operating in *Shewanella* and *Geobacter* species.

### *c*-type cytochrome-based extracellular electron transfer in thermophilic Gram-positive organisms

**Fig. 3:**
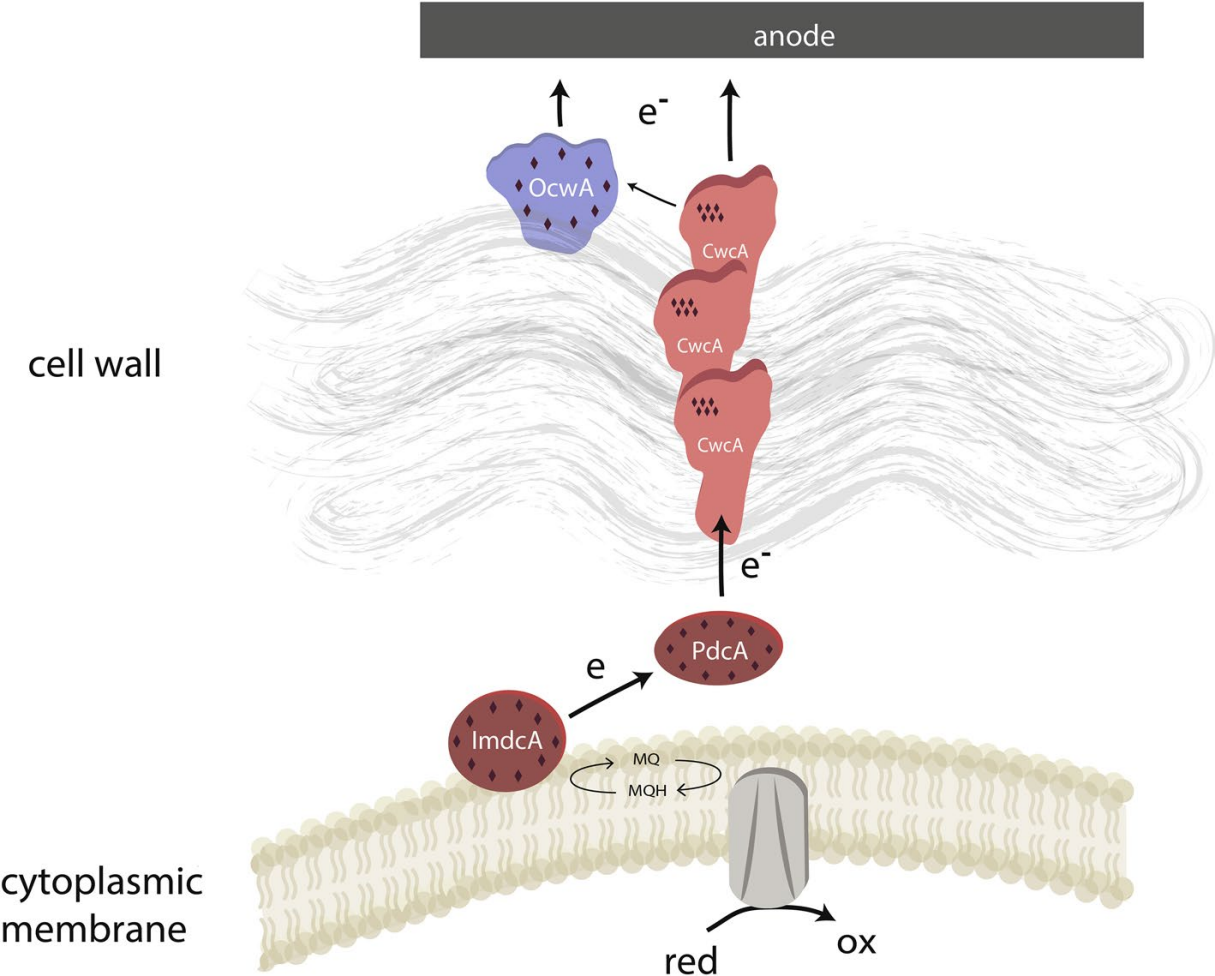
Scheme of the extracellular electron transfer pathway in *T. ferriacetica*. Diamonds indicate the number of heme cofactors in the respective *c*-type cytochromes. Scheme modified from Faustino et al. (2021)

The best understood model organisms for thermophilic electron transfer onto anode surfaces are the two closely related organisms *Thermincola ferriacetica* and *Thermincola potens. T. ferriacetica* was isolated in 2007 from a terrestrial hydrothermal spring and characterized as a chemolithoautotrophic ferric iron reducing spore forming organism (Zavarzina et al. 2007). Studies conducted with mixed cultures in microbial fuel cells operated at higher temperatures revealed organisms belonging to the genus *Thermincola* as key organisms which is why *T. ferriacetica* was later successfully applied as anode reducing organism in a bioelectrochemical system (Mathis et al. 2008; Fu et al. 2013). In a parallel study, another strain of the genus was directly isolated from a microbial fuel cell inoculated with a sample from a thermophilic methanogenic anaerobic digester and operated at 56 °C. The isolate was named *Thermincola potens* JR1 (Wrighton et al. 2011). The two strains share 99% 16S rRNA sequence identity and they seem to share the same strategy for ferric iron reduction. Nevertheless, they behave differently in bioelectrochemical systems. *T. ferriacetica* can form rather thick biofilms that can grow up to more than 150 µm thickness, while *T. potens* biofilms are rather thin and reach even after one month of operation a thickness of only about 20 µm (Wrighton et al. 2011; Parameswaran et al. 2013; Lusk et al. 2016). Of note, the current density of *T. potens* biofilms after one month of operation did not change compared to the current density produced by initially formed monolayers on the anode surfaces. Hence, further biofilm layers apparently did not participate in current production. This leads to the conclusion that the biofilm is not conductive and only direct cell electrode interaction leads to current production. This situation is different in *T. ferriacetica* biofilms that seem to be conductive and therefore allow cells in distance of the electrode to participate in electron transfer as well. Nevertheless, fundamental knowledge on the mechanism of generating a conductive biofilm matrix by *T. ferriacetica* is largely missing. Still, life dead staining analysis revealed viable cells of *T. ferriacetica* up to 25 µm away from the anode surface and a maximum current density of up to 12 A m^−2^ was reached in a microbial electrolysis cell with an anode poised to a potential of -0.06 V vs SHE (Parameswaran et al. 2013). First evidence regarding the mechanism of electron transfer came from a study by Carlson and colleagues (Carlson et al. 2012). The authors used cells grown with ferric iron as electron acceptor and studied the identity of cell surface proteins as well as their localization by denaturant extraction and trypsin shaving. The results indicated that the hexaheme protein Ther_JR_1122 (CwcA) seems to be localized within the cell wall of the organism while the two decaheme cytochromes Ther_JR_1117 (ImdcA) and Ther_JR_0333 (PdcA) are localized at the cytoplasmic membrane and the nonaheme Ther_JR_2595 (OcwA) on the surface of the cell. Hence, similar to *Shewanella* and *Geobacter c*-type cytochromes bridge the gap between cytoplasmic membrane and surface of the extracellular electron acceptor (Fig. 3). The challenge of bridging the distance through the multilayer cell wall could be overcome by formation of polymeric structures of CwcA similar to OmcS in *G. sulfurreducens*. Evidence, for the latter stems from bioinformatic homology modeling of CwcA along the polymeric structure of OmcS and the fact that it was not possible to stabilize the protein in a soluble form in a purification attempt (Faustino et al. 2021). The midpoint redox potentials of the heme proteins are overlapping and are in a range between + 100 mV and − 300 mV vs SHE. The strategy of an electron transfer chain composed of *c*-type cytochromes with overlapping potentials evolved in parallel in Gram-negativ organisms like *S. oneidensis* and *G. sulfurreducens*. Direction of electron flow is consequently almost solely dependent on the redox potential gap between the quinol reductase and the terminal electron acceptor.

### Bioinformatic evidence for the distribution of extracellular electron transport pathways in extremophiles

Light and colleagues (2019) identified homologous genes in several species within the firmicutes for a potential flavin-based EET. These include the thermophiles *Eremococcus coleocola, Ignavigranum ruoffiae, Lactococcus garviae, Amphibacillus jilinensis, Caldanaerobius fijiensis, Thermanaerobacter mathranii, Mahella australiensis*, the haloalkalophiles *Alkalibacterium gilvum, Oceanobacillus oncorynchi* and psychrophilic *L. lactis*.

An equally comprehensive approach for *c*-type cytochrome-based EETs conducted here results in an unmanageable number of organisms. But focusing only on extremophilic organisms, we could identify species of 12 genera bearing homologous genes for one of the three beforementioned pathways from *S. oneidensis, G. sulfurreducens* and *T. potens*.

**Fig. 4:**
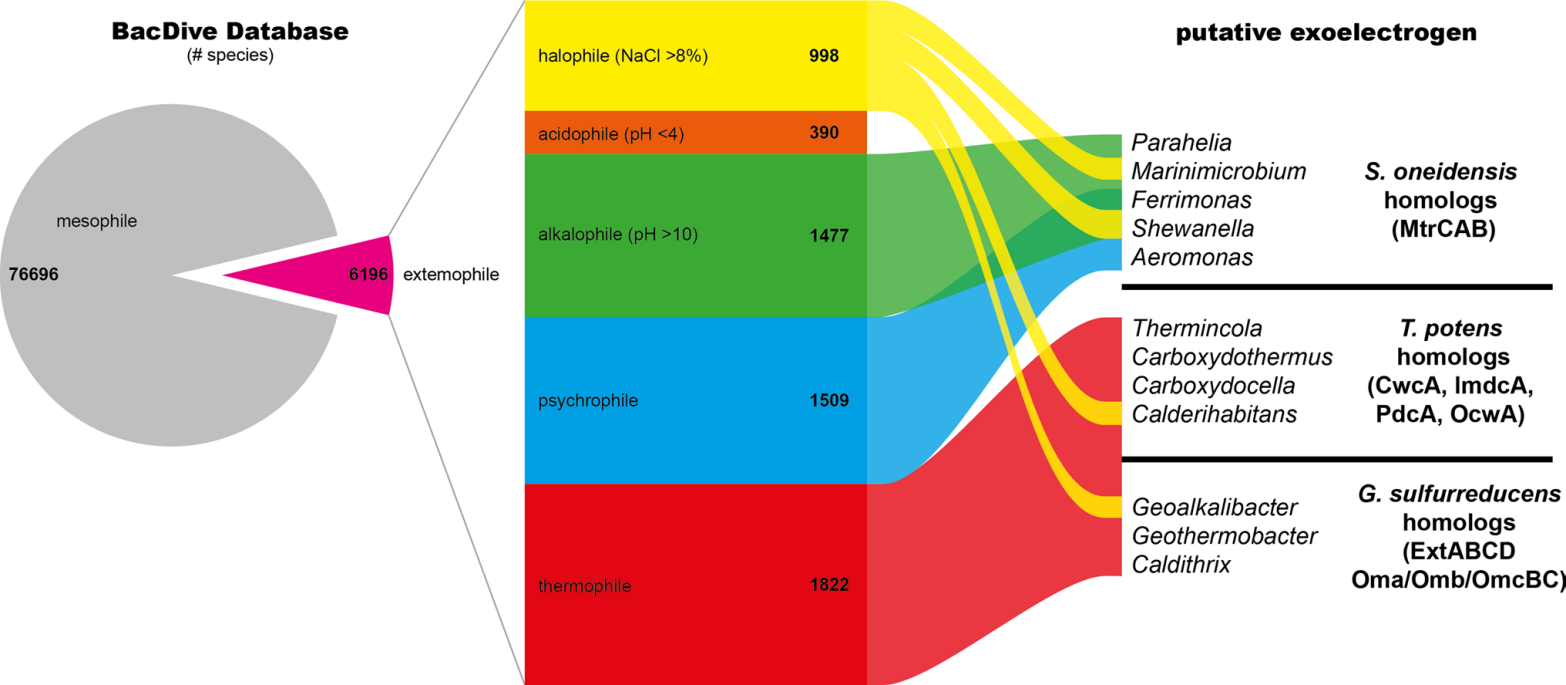
Extremophiles in the BacDive database and genera carrying genes for *c*-type cytochrome-based EET genes. 7.5% of species listed in the BacDive database are considered as extremophilic. Within these, species of 12 genera carry genes for EET, which are grouped on the righthand side

Based on the BacDive database (Reimer et al. 2022), species were selected which are described as psychrophilic, (hyper) thermophilic, show growth at pH below 4 and above 10 or at NaCl concentration above 8% (w/v). Of those, genome-based protein-sequences were recovered from the UniProt database (UniProt Consortium 2021) and used as a target database for a BlastP of EET key genes. Nearly 7% of species listed in BacDive are extremophiles (Fig. 4). There seems to be a clear division, how EET are used in different environments. Alkaliphilic or halophilic organisms seem to use the Mtr-pathway, while thermophiles carry homologues to *Geobacter* genes (Gram-negative) or to *Thermincola* genes (Gram-positive). For acidophiles no EET pathways could be predicted. The latter correlates well with the high solubility of electron acceptors like ferric iron at low pH compared to almost complete insolubility at neutral conditions.

## The biochemistry of microbial electron import from cathodes

Limited mechanistic information is currently available regarding the transport of electrons from a cathode into a microbial cell (Igarashi and Kato 2017; Kato 2016; Logan et al. 2019). In fact, evidence for a direct electron import from a cathode is so far only available from studies dealing with the reversibility of *c*-type cytochrome-based electron transport chains from *S. oneidensis* and *G. sulfurreducens* (Tang et al. 2019). For example, anodically precultured *S. oneidensis* biofilms can accept cathodic electrons with a negative potential of − 360 mV vs. SHE for the reduction of fumarate after electrode repolarization (Ross et al. 2011). Also, the reduction of oxygen by cathodic *S. oneidensis* biofilms could be observed at a potential of − 300 mV vs. SHE (Rowe et al., 2018). Previously, uptake of cathodic electrons coupled to reduction of fumarate has also been demonstrated in several *Geobacter* species (Dumas et al. 2008).

**Fig. 5:**
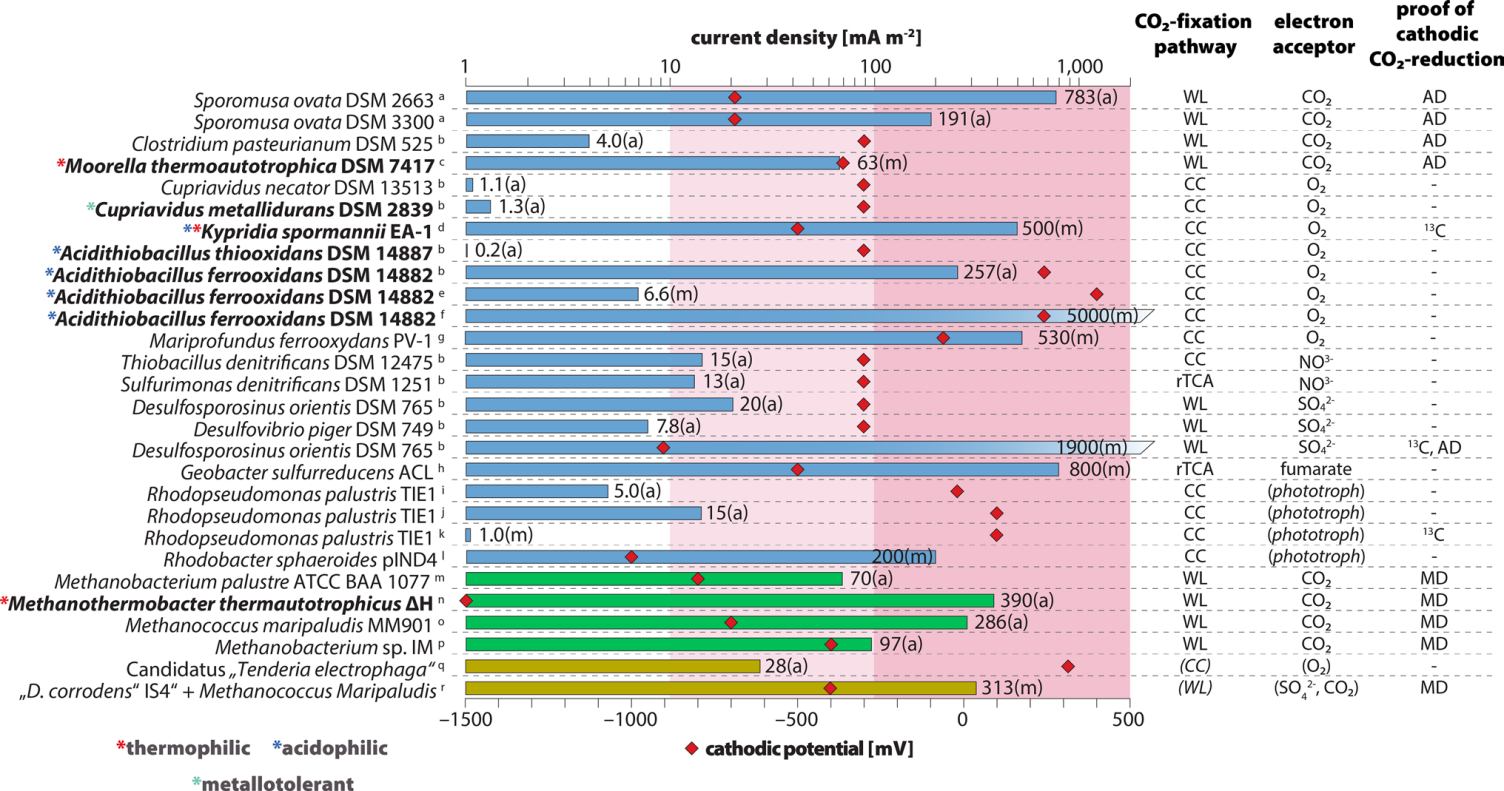
Cathodic current consumption of electroactive, autotrophic organisms, the corresponding CO_2_-fixation pathway and electron acceptor, and the applied method of cathodic CO_2_-fixation evidence. Care was taken in the selection of studies to ensure that the cathode served as the sole electron source for CO_2_-reduction. Extended and modified from Logan (Logan et al. 2019). Bar length corresponds to average (a) or maximum (m) current density. Cathodic potential is indicated by the position of the red diamond. Blue bars are associated with Bacteria, green bars were chosen for Archaea. Yellow bars refer to mixed cultures. *WL* Wood-Ljungdahl pathway, *CC* Calvin cycle, *rTCA* reductive citrate cycle, *AD* acetate detection, *MD* methane detection, *13C* proof of CO_2_ fixation by isotope analyses. a: Aryal et al. (2017); b: de Campos Rodrigues and Rosenbaum (2014); c: Yu et al. (2017); d: Reiner et al. (2020); e: Ishii et al. (2015); f: Carbajosa et al. (2010); g: Summers et al. (2013); h: Ueki et al. (2018); i: Doud and Angenent (2014); j: Bose et al. (2014); k: Guzman et al. (2019); l: Schmid et al. (2021); m: Cheng et al. 2009; n: Sato et al. (2013); o: Lohner et al. (2014); p: Beese-Vasbender, Grote, et al. (2015); q: Wang et al. (2015); r: Deutzmann and Spormann (2016)

Electron transport chains from the cell surface to the cytoplasmic membrane are not uncommon in microorganisms. Ferrous iron, Fe^0^ as well as molecular sulfur belong to widely distributed electron donors which are typically oxidized at the cell surface. In fact, modules similar to the MtrAB complex of *S. oneidensis* were found in the genomes of different iron(II)-oxidizing microorganisms. An example are PioA and PioB, essential for phototrophic iron(II) oxidation in the purple bacterium *Rhodopseudomonas palustris* TIE1. Nevertheless, ferrous iron oxidizing organisms usually thrive with dissolved Fe^2+^. This might be the reason why *R. palustris* or the chemolithotrophic iron oxidizer *Siderooxydans lithotrophicus* have a *c*-type cytochrome-based electron transfer chain starting with proteins similar to MtrA and MtrB but lacking a cell surface localized protein like MtrC, which could interact with a cathode as surrogate for an insoluble electron donor. Nevertheless, *R. palustris* TIE1 belongs to the few organisms for which some experiments point towards potential electron uptake from a cathode surface (Fig. 5). Still, *c*-type cytochrome dependent electron import does not necessarily depend on modules similar to MtrAB. An example is the acidophilic ferrous iron oxidizing microorganism *Acidithiobacillus ferrooxidans*, which will be mentioned in more detail below.

A direct extracellular electron transport was also postulated for microorganisms responsible for microbial induced corrosion (MIC) of elemental iron (Venzlaff et al. 2013). Diverse microorganisms of different physiological groups are capable of this oxidation of Fe (0). Especially representatives of sulfate-reducing and acetogenic bacteria, but also methanogenic archaea are attributed with the ability to MIC (Beese-Vasbender et al. 2015; Deutzmann and Spormann 2016; Kato 2016; Logan et al. 2019). However, although most of these organisms in a BES can also use cathodic electrons as electron donors, the molecular mechanisms of electron uptake are so far not clear (Kato 2016). The majority of electroautotrophic microorganisms described in literature at this time are not capable of catalyzing respiratory iron oxidation, but can use molecular hydrogen as a respiratory electron donor. Consequently, electron uptake mediated by cellular hydrogenases, has been proposed as potential mechanism of direct EET (Mohanakrishna et al. 2016; Rosenbaum and Franks 2013; Rosenbaum et al. 2011). However, microbially catalyzed hydrogen evolution on cathodes is a highly debated research field and indirect hydrogen-based EET cannot be excluded from a thermodynamic point of view in many studies. It has been shown that a biocathode can significantly lower the overpotential of the hydrogen evolution reaction even though the exact mechanisms of this catalysis are unclear. In addition, the cathodic potentials at which hydrogen evolution can be observed differ greatly. Yates and colleagues were able to demonstrate hydrogen evolution on a biocathode starting at a cathodic potential of − 600 mV vs SHE (30 °C; pH 6.8). Killing the cells with ethanol did not lead to a drastic change in hydrogen production (Yates et al. 2014). Thus, it can be assumed that the reduction of the overpotential in this case is not catalyzed by physiological processes but due to an electrode activation by some inorganic or organic components of the microbial culture. Using the sulfate-reducing MIC bacterium *Desulfopila corrodens* IS4 as a biocatalyst in a BES, Deutzmann and colleagues were able to demonstrate hydrogen production already at a potential of − 400 mV vs SHE (30 °C; pH7) (Deutzmann and Spormann 2016). At standard biological conditions (pH 7), the redox potential of hydrogen production from electrons and protons is − 413 mV vs SHE. However, at low hydrogen partial pressure (due to immediate microbial hydrogen oxidation) and low pH, the redox potential of this proton-dependent reaction can reach much higher values (e.g., − 89 mV vs SHE at pH 2 and a hydrogen partial pressure of 10^–5^ atmospheres). Besides the pH the temperature is also crucial for the required hydrogen generation potential. According to Steven Bratsch, a difference in potential of 0.38 mV/K can be assumed in aqueous solutions (Bratsch 1989). In addition to abiotic and biomass-catalyzed hydrogen evolution, Deutzmann and colleagues also identified enzyme-based production of reduced compounds, such as hydrogen or formate, by free, extracellular hydrogenases and formate dehydrogenases as another mechanism of indirect EET from a cathode (Deutzmann et al. 2015; Lienemann et al. 2018; Lohner et al. 2014). This enzyme-based catalysis of mediated electron transfer is likely due to a partial cell lysis during cathodic cultivation.

In summary, so far two mechanisms have been discovered that allow for electron import. First, the direct import of electrons via *c*-type cytochrome-based electron transfer machineries and second an indirect electron import that is mediated via abiotic or biotic catalysis of hydrogen or formate evolution.

## Extremophilic organisms thriving on cathodes

So far, there is only a limited number of pure cultures from extremophilic organisms for which it was proven that the cathode is the only electron source for microbial metabolism. Moreover, there is only one organism for which active growth on the cathode was proven so far.

Three different studies reported on the bioelectrochemical activity of the acidophilic strain *A.ferrooxidans* (de Campos Rodrigues and Rosenbaum 2014; Ishii et al. 2015; Chabert et al. 2018). The organism is a chemolithoautotrophic ferrous iron oxidizer. The electron transport chain from ferrous iron to oxygen is *c*-type cytochrome dependent. While a cell surface localized cytochrome *c2* catalyzes ferrous iron oxidation, rusticyanin as well as a cytoplasmic membrane localized cytochrome transfer electrons electrochemically downhill towards the *aa3* complex which is the cellular side for oxygen reduction and hence, proton motif force (PMF) generation. Rusticyanin seems to be the branching point for electron transfer as it was hypothesized that transfer from rusticyanin towards the cytochrome *bc1* complex marks the core of the PMF dependent uphill electron transport pathway used for NADH production. Ishii and colleagues conducted inhibitor studies using *A.ferrooxidans* cells thriving on a cathode surface. These experiments suggest that the organism uses the same above-described pathways for ferrous iron as well as cathode oxidation. Along these lines, Chabert and colleagues could increase bioelectrochemical efficiency of the organism by adding quorum sensing messenger molecules in an initial biofilm formation phase (Chabert et al. 2018). The addition of a mixtures of homoserine lactones increased surface coverage and led to higher reductive currents compared to control experiments. Interestingly, a comparative study with the two phylogenetically related strains *A.ferrooxidans* and *A. thiooxidans* that differ in that *A. thiooxidans* cannot oxidize iron revealed that the latter cannot catalyze the production of significant cathodic currents (de Campos Rodrigues and Rosenbaum 2014). This is further support for the hypothesis that the iron oxidation pathway is also used for electron import from cathodes.

The methanogen *Methanothermobacter thermoautotrophicus* as well as the acetogen *Moorella thermoautotrophica* were both studied as electroautotrophic organisms on cathodes (Sato et al. 2013; Yu et al. 2017). As both organisms are hydrogen-consuming it is as mentioned above not easy to experimentally proof electron import that is independent on hydrogen production on the electrode surface. Nevertheless, while *A. ferrooxidans* uses cathodic electrons for energy and biomass production producing water as side product, the conducted analyses of *M. thermoautotrophica* thriving on the cathode delivered clear benchmarks for cathode dependent thermophilic acetate and formate production. At 55 °C and a cathode potential of − 400 mV vs SHE, the authors revealed production rates of 58.2 and 63.2 mmol m^− 2^ day^− 1^ at a coulombic efficiency of 65% for formate and acetate, respectively (Yu et al. 2017).

Among the tested organisms in a screen for electrotrophic organisms were also metal-resistant knallgas bacteria like *Cupriavidus necator* or *C. metallidurans* (de Campos Rodrigues and Rosenbaum 2014). Nevertheless, no significant electron uptake activity could be detected. This is in line with a study by Li and colleagues, which suggested that direct interaction of *Cupriavidus* strains with a cathode is hampered by reactive oxygen species produced at the cathode or anode due to either incomplete water oxidation or oxygen reduction, respectively (Li et al. 2012).

Nevertheless, Reiner and colleagues were able to isolate a thermophilic knallgas bacterium that can thrive on cathode surfaces poised to a potential of − 350 mV vs SHE (Reiner et al. 2018, 2020). The inoculum for isolation originated from a mixture of hot spring samples from the Azorean Islands, Portugal. Moreover, the authors delivered the first proof for active growth of a pure culture on a cathode surface. The organism *Kyrpidia spormannii* grows at 60 °C and pH 3 on plain graphite electrodes and builds biofilms of up to 100 µm thickness as analyzed by optical coherence tomography analysis (Hackbarth et al. 2020). The organism uses the Calvin cycle for CO_2_ fixation and is a natural producer of the storage polymer polyhydroxybutyrate, which is applied as bioplastic material. It is so far not entirely clear how the organism is able to use the cathode as electron donor. As the most likely mechanism might be the consumption of hydrogen which is produced even at rather high potentials at pH 3, selective evolution experiments also suggested that the ability of the organism to grow also with molecular sulfur as electron donor might have an impact. Jung and colleagues grew the organism for several transfers on cathodes and observed a fourfold increase of the biofilm accumulation rate (Jung et al. 2021). Bioinformatic analysis revealed that the adapted cells carried three loud mutations compared to the progenitor strain. These mutations occurred in the genes for a DNA-repair protein, a regulator for response to oxidative stress as well as a gene for a subunit of a CoB-CoM heterodisulfide reductase. Further analysis revealed that the organism cannot only grow with hydrogen as lithotrophic electron donor but also molecular sulfur and that this ability is slightly advanced in the selectively evolved organism. Still, as a genetic system is not available for the organism thus far, direct proof for a potential influence of the electron transport chain from sulfur in electron import from cathodes is still missing. Nevertheless, that the production of reactive oxygen species on the electrodes also hampers the activity of *K. spormannii* is rather undoubtedly expressed by the mutation within the *perR* regulator gene which will likely lead to constitutive production of proteins dealing with oxidative stress (Jung et al. 2021). Moreover, Pillot et al. recently modified graphite cathodes by means of electrodeposition with different catalytically active metals such as platinum that enhance abiotic hydrogen evolution. This modification had only little effect on the cell number of cathodic *K. spormannii* biofilms grown for three days. Although these results might be indicative of a hydrogen-independent electron uptake, it remains unclear whether the likewise improved oxygen reduction capability of the modified cathodes led to an elevated production of reactive oxygen species that hindered cathodic biofilm growth (Pillot et al. 2022).

## Future research direction

Past research taught us that several biochemical solutions for electron exchange between organisms and cathodes or anodes exist. Especially, in the microbe-anode-interaction field we can observe an interesting bias of certain solutions for extracellular electron transfer occurring more often in some extremophilic regimes than in others. The reasons for this have so far not been elucidated. Also, in the last years we observed that there are way more mechanisms for extracellular electron transfer compared to what was believed so far. To find something new in this field demands the work of microbiologists enriching and isolating new organisms with the ability to interact with solid electron donors and acceptors.

Bioelectrochemistry holds great promise regarding an application in the fields of environmental engineering, biotechnology or bioelectronics. It will be interesting to observe in the future if potentially more stable electron transferring proteins from extremophiles will find their way into new forms of electronic devices. Moreover, the field of steered development of conductive biofilms to increase space time yields in bioelectrochemical systems becomes more and more momentum. In this regard, Philipp and colleagues calculated necessary current densities that have to be reached in order to achieve bioelectrochemistry based production of chemicals with competitive space time yields (Philipp et al. 2020). Last but not least, we have to develop new reactor technologies to exploit the abilities of exoelectrogenic microorganisms. This will become particularly important in the field of thermophilic organisms thriving on cathodes as these organisms could be excellent biocatalyst for the sustainable upcycling of hot industrial off-gases to platform chemicals.
